# A practical generalization metric for deep networks benchmarking

**DOI:** 10.1038/s41598-025-93005-5

**Published:** 2025-03-21

**Authors:** Mengqing Huang, Hongchuan Yu, Jianjun Zhang

**Affiliations:** National Centre for Computer Animation, Bournemouth University, Poole, BH12 5BB UK

**Keywords:** Computer science, Information technology

## Abstract

There is an ongoing and dedicated effort to estimate bounds on the generalization error of deep learning models, coupled with an increasing interest with practical metrics that can be used to experimentally evaluate a model’s ability to generalize. This interest is not only driven by practical considerations but is also vital for theoretical research, as theoretical estimations require practical validation. However, there is currently a lack of research on benchmarking the generalization capacity of various deep networks and verifying these theoretical estimations. This paper aims to introduce a practical generalization metric for benchmarking different deep networks and proposes a novel testbed for the verification of theoretical estimations. Our findings indicate that a deep network’s generalization capacity in classification tasks is contingent upon both classification accuracy and the diversity of unseen data. The proposed metric system is capable of quantifying the accuracy of deep learning models and the diversity of data, providing an intuitive and quantitative evaluation method - a trade-off point. Furthermore, we compare our practical metric with existing generalization theoretical estimations using our benchmarking testbed. It is discouraging to note that most of the available generalization estimations do not correlate with the practical measurements obtained using our testbed. On the other hand, this finding is significant as it exposes the shortcomings of theoretical estimations and inspires new exploration.

## Introduction

Generalization refers to a model’s ability to perform well on unseen or new data, emphasizing its capacity to capture underlying patterns in the data rather than merely memorizing specific details from the training set. A well-generalized model not only excels on the training data but also demonstrates strong performance on previously unseen data. The assessment of generalization in deep networks has predominantly focused on supervised learning settings.

Currently, while efforts to establish theoretical bounds for generalization continue, there is growing interest in intuitive metrics for experimentally assessing generalization capacity. This trend reflects concerns that many theoretical bounds or capacity measures may be vacuous, inefficient, or even counterproductive in practice. Recent studies have explored various properties associated with deep network generalization. For instance, research has focused on robust overfitting in adversarial training^1^, distributional robustness as a measure of generalization, and combining complexity measures^2^. Additionally, there is ongoing inquiry into whether causal relationships between these complexity measures and generalization can be accurately identified^3^. Recent advancements in the estimation of non-vacuous generalization bounds^4^ have proposed approaches for constructing tighter bounds, aiming to better elucidate the relationship between data fit and model compression. However, these theoretical estimations require practical validation, as well as a benchmarking framework for evaluation and comparison. The field of deep learning generalization has also been enriched by a growing consensus that traditional machine learning theory, grounded in worst-case analyses, is insufficient to fully explain the generalization capabilities of deep learning models^5^. This is particularly true when trying to understand why over-parameterized neural networks often generalize well^6^. For instance, Dupuis et al.^7^ introduced a data-dependent fractal dimension to enhance generalization bound estimations, while Neyshabur et al.^6^ proposed a complexity measure based on unit-wise capacities, yielding more precise bounds for two-layer ReLU networks. Furthermore, Valle-Pérez et al.^8^ reviewed generalization error bound estimation methods, proposing seven desiderata for evaluating generalization in deep learning models and systematically categorizing existing approaches based on these criteria.

Generalization bounds can be broadly classified into four categories. The first category, data-independent and algorithm-independent, includes algorithms with minimal assumptions, such as VC dimension bounds^9^. The second category, data-dependent and algorithm-independent, relies on training data and includes approaches like the Rademacher complexity bound^10,11^. The third category, data-independent and algorithm-dependent, incorporates stronger assumptions without depending on training data^12–14^. Lastly, the data-dependent and algorithm-dependent category includes methods that make strong assumptions and depend on training data, such as those presented in^15–22^. Notably, Dziugaite et al.^23^ introduced the first non-vacuous PAC-Bayes generalization bounds for deep stochastic neural networks on the binary MNIST dataset, and subsequent work^4^ proposed novel compression techniques for constructing tighter bounds.

In addition to theoretical advancements, empirical studies suggest that model size plays a more critical role in generalization than specific architectural details, such as network width or depth. For instance, Kaplan et al.^24^ demonstrated that neural scaling laws indicate a strong correlation between model or dataset size and performance, a finding supported by van Rossem et al.^25^ and Huang et al.^26^, who showed that certain behaviors remain consistent across architectures once models are sufficiently flexible. This universality suggests that architectural specifics have a minimal impact on the learned representations.

Moreover, the Predicting Generalization in Deep Learning competition^27^ at NeurIPS 2020 highlighted the need to understand the relationship between model complexity and generalization. The competition featured eight tasks with pre-trained deep networks of similar architectures but differing hyperparameters. Conditional Mutual Information was applied to explore these relationships. While our proposed metric does not directly compute model complexity, it captures dimensions related to robustness and model size, aiming to provide a broader perspective on hyperparameter variations and generalization gaps.

This paper introduces a practical metric, the trade-off point approach, for measuring generalization capacity and proposes a novel benchmark testbed for evaluating various deep networks. Our observations suggest that a deep network’s generalization capacity in classical classification scenarios depends on both classification accuracy and the diversity of unseen data. The proposed testbed quantifies both model accuracy and test data diversity, offering an intuitive and quantitative method for assessing generalization.

In addition, compared to existing complexity measures^2^, our proposed metric focuses on models with varying architectures for both comparison and benchmarking, rather than a single model solely for benchmarking. Nonetheless, our benchmark testbed allows for the comparison and assessment of existing complexity measures. Our findings indicate that most complexity measures do not align with practical measurements, raising questions about the validity of current theoretical generalization estimations. The main contributions of this paper include:

- Introducing a practical generalization metric for comprehensively benchmarking deep networks.
- Verifying theoretical generalization estimations through the proposed benchmark testbed.

## Methods

**Fig. 1:**
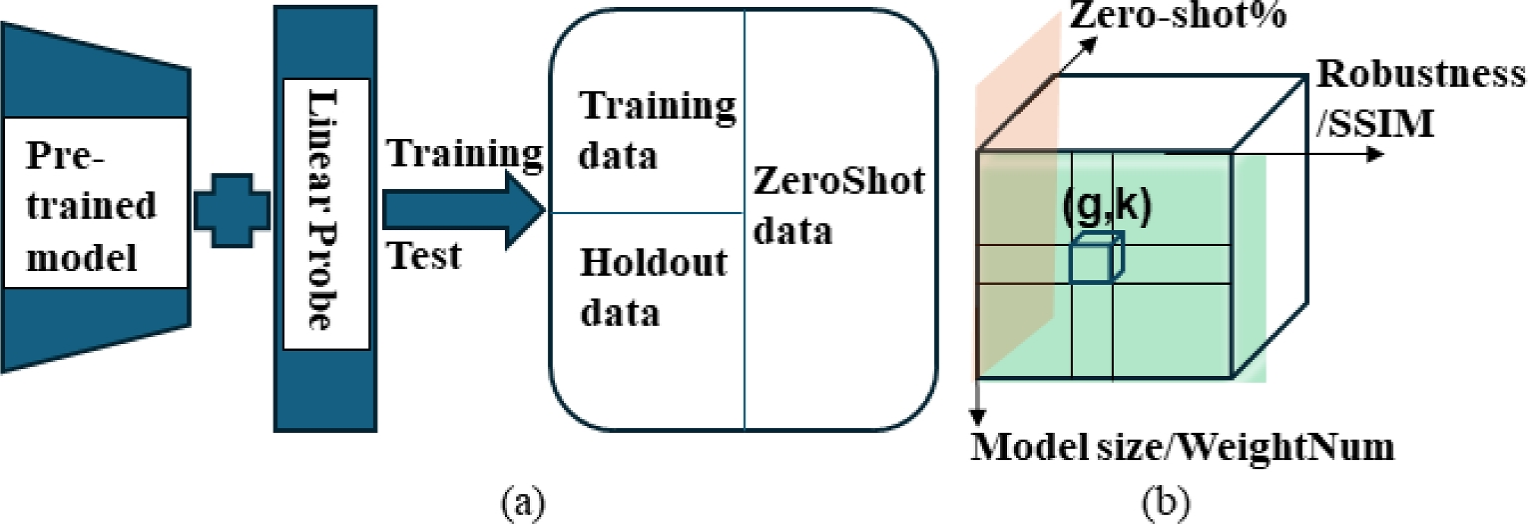
(**a**) Illustration of benchmark testbed; (**b**) a 3D array consists of cells (*g*, *k*), and the pink piece refers to the slice without noise (SSIM = 1) and blue piece refers to the slice with zero-shot% = 0.

The proposed metric is to measure the generalization capacity of a model through the accuracy (such as classification correct or error rates) and the diversity of test data (such as Kappa) in terms of three factors (i.e. model size, robustness, zero-shot data). Our framework for benchmarking the generalization of deep networks comprises two integral components: the Benchmark Testbed, responsible for producing raw data, and the practical Generalization Metric, which evaluates the model’s generalization capacity.

### Benchmark testbed

The proposed benchmark testbed utilizes the linear probe CLIP structure^28^ to evaluate how effectively a deep learning model captures essential features within its hidden layers. Specifically, this involves training a simple linear model, such as logistic regression, on a designated training dataset to adapt the tested models to the specified task. All tested models are pretrained and combined with the linear probe in our implementation.

Notably, since the linear probe cannot capture complex patterns, high performance indicates that the complexity resides in the features themselves rather than in the linear probe. Figure 1a illustrates the Benchmark Testbed. Here, the pretrained model, adapted with a linear probe, is trained on specific training data and subsequently evaluated on holdout data to assess its performance.

Experimentally, the data is divided into two parts: the training data and the holdout data, both sharing the same classes. The pre-trained models are fine-tuned on the training dataset and then tested on the holdout dataset. We gather measured data, specifically ErrorRate and Kappa (defined by Eqs. 1 and 2), across three distinct dimensions: model size (representing the number of weights), robustness (adding noise and using Structural Similarity Index as a metric, SSIM), and zero-shot capacity (using the percentage of unseen classes).

Notably, the model size dimension is an important factor. “Scaling law”^29^ has pointed out that the model performance depends on the model size, training dataset size and amount of compute used for training. Although model size does not precisely reflect the architecture of the tested models, training dataset size and amount of compute used for training, it serves as an important indicator for benchmarking purposes.

Regarding the robustness dimension, in deep learning, robustness measures how well a network performs under controlled variations such as noise or distortions, providing insights into the network’s ability to generalize effectively^30^. This concept is extended to adversarial robust learning settings under the umbrella of adversarial robustness. Recent works focus on the generalization gap in robust learning contexts^31,32^. Li et al.^33^ and Kim et al.^34^ further explore robust generalization challenges in adversarial learning models. Moreover, Bubeck et al.^35^ highlights that “over-parameterization” is also necessary for robust learning. Consequently, robustness is incorporated into our testbed by introducing adversarial samples into the test data.

We use the percentage of unseen classes in the data as the zero-shot dimension to assess zero-shot capacity. It is reasonable that when applying the fine-tuned tested models to the zero-shot data, the percentage of unseen classes in the data serves as an indicator of zero-shot capacity.

This approach results in a three-dimensional array, as shown in Fig. 1b. Each cell within this array records the distributions of ErrorRate (denoted as “g”) and Kappa metrics (denoted as “k”) across all classes. Different cells within the 3D array correspond to individual settings of the three dimensions. This comprehensive evaluation procedure offers insights into the efficacy of feature extraction within the pre-trained model, allowing an assessment of how well these captured features generalize to new or unseen data.

The generalization gap is defined by Jiang et al.^27^,

1 $$\begin{aligned} \begin{aligned} g\left( f_w ; D\right) =\frac{1}{\left| D_{\text{ test } }\right| } \sum _{(x, y) \in D_{\text{ test } }} \mathbbm {1}\left( f_w(x) \ne y\right) -\frac{1}{\left| D_{\text{ train } }\right| } \sum _{(x, y) \in D_{\text{ train } }}\mathbbm {1}\left( f_w(x) \ne y\right) \end{aligned} \end{aligned}$$

where *w* denotes a set of model’s weights. Moreover, various hyperparameter types introduce diverse weight values, which results in many variations of some model. Ideally these variations inherit properties of the original model. A rising issue is to capture changes in every single hyperparameter type and measure changes in generalization gap accordingly. In an effort to replicate this random space, Jiang et al.^27^ selects weight values from a spectrum of hyperparameter types. However, we have another opinion, that is, the variations of some model may be regarded as different models. This is because they may have individual network connection, layers, weights etc. If they are regarded as individuals, our benchmark testbed can test these variations in-depth and streamline model design accordingly.

### Practical generalization metric

The proposed metric is to seek for a trade-off point to illustrate the generalization of test models as follows.

Step 1: We compute the ErrorRate of individual classes on the test data using Eq. (1). It enables the derivation of a distribution of error rates across all classes, while the generalization error typically refers to the overall error rate. We then evaluate the diversity of the test data using the Kappa statistic^36^. In the context of multi-class classification problem, we are dealing with agreement and disagreement among classifier outputs. The Kappa is indeed more robust than simple percentage agreement because it adjusts for the possibility of agreement occurring by chance^37^. This is particularly useful when there is a class imbalance, as chance agreement would be higher for the more frequent classes^38^. To highlight diversity issue, we design a Kappa on a specific class to result in a distribution of Kappa across all classes.

The confusion matrix for multiclass classification^38^ is defined as,

$$\begin{array}{|c|c|c|c|c|} \hline \text {Ground truth Estimated} & C_1 & \cdots & C_m & \text {Row margin} \\ \hline C_1 & n_{1,1} & \cdots & n_{1,m} & n_{1,.} = \sum _{j=1}^{m} n_{1,j} \\ \hline \vdots & \vdots & \ddots & \vdots & \vdots \\ \hline C_i & n_{i,1} & \cdots & n_{i,i} & n_{i,.} = \sum _{j=1}^{m} n_{i,j} \\ \hline \vdots & \vdots & \ddots & \vdots & \vdots \\ \hline C_m & n_{m,1} & \cdots & n_{m,m} & n_{m,.} = \sum _{j=1}^{m} n_{m,j} \\ \hline \text {Column margin} & n_{.,1} = \sum _{i=1}^{m} n_{i,1} & \cdots & n_{.,m} = \sum _{i=1}^{m} n_{i,m} & N = \sum _{i=1}^{m} \sum _{j=1}^{m} n_{i,j} \\ \hline \end{array}$$

where m denotes the class number, N denotes the total sample number. The probabilities can be estimated as,

$$p_{i,j} = \frac{n_{i,j}}{N}$$

We hope to see the classifier’s performance change across all classes. Thus, the confusion matrix is redefined for a specific class as follows. Herein, the sample set is divided into two parts, i.e., *i*-th class and non-*i*-th classes, $\overline{i}$-th classes.

$$\begin{array}{|c|c|c|c|c|} \hline \text {Ground truth Estimated} & C_i & \overline{C}_i & \text {Row margin} \\ \hline C_i & p_{i,i} & p_{i,\overline{i}} & p_{i,.} = p_{i,i} + p_{i,\overline{i}} \\ \hline \overline{C}_i & p_{\overline{i},i} & p_{\overline{i},\overline{i}} & p_{\overline{i},.} = p_{\overline{i},i} + p_{\overline{i},\overline{i}} \\ \hline \text {Column margin} & p_{.,i} = p_{i,i} + p_{\overline{i},i} & p_{.,\overline{i}} = p_{i,\overline{i}} + p_{\overline{i},\overline{i}} & \\ \hline \end{array}$$

where $$p_{i,\overline{i}} = \sum _{j \ne i} p_{i,j}, \quad p_{\overline{i},\overline{i}} = \sum _{j \ne i} \sum _{k \ne i} p_{jk}.$$

The Kappa about the i-th class is defined as,

2 $$\begin{aligned} \left\{ \begin{gathered} k_i=\frac{P_0-P_e}{1-P_e}\\ P_0= p_{i,i}+p_{\overline{i},\overline{i}} \\ P_e=p_{i,.}p_{.,i}+p_{\overline{i},.}p_{.,\overline{i}} \end{gathered} \right. \end{aligned}$$

The average of the Kappas for all the classes may be regarded as the generalization Kappa. A model with strong generalization capacity should be adaptable to highly diverse data. When the Kappa statistic is high, it indicates that the model exhibits high diversity, and has a high generalization capacity.

Step 2: Within the three dimensions (zero-shot%, weight number, robustness) of the 3D array, we can calculate two distributions on a cell-wise basis: one related to ErrorRate and the other to Kappa. These calculations are carried out by Eq. (1) for ErrorRate and Eq. (2) for Kappa, and are stored within the 3D array (denoted as a pair of “*g* and *k*” for each cell, see Fig. 1b).

We depict these two distributions of each cell by three kinds of statistics, i.e., means (denoted as *M*), standard deviations (denoted as *SD*), and 10*th* percentiles (denoted as $^{10}P$). The 10*th* percentile score indicates that 10% of the trials scored below it. Since smaller means are better in this context, the 10*th* percentiles represent the best performing 10% of classification outcomes.

We update each cell in the 3D array by these three kinds of statistics with respect to two distributions (i.e., ErrorRate and Kappa) within three dimensions, that is, $M_{g}(ZeroShot, Robust,WeightNum)$, $SD_{g}(ZeroShot, Robust,WeightNum)$, $^{10}P_{g}(ZeroShot, Robust,WeightNum)$ on ErrorRate and $M_{k}(ZeroShot, Robust,WeightNum)$, $SD_{k}(ZeroShot, Robust,WeightNum)$, $^{10}P_{k}(ZeroShot, Robust,WeightNum)$ on the updated Kappa (i.e. {1-k_i_}, i=1..m). Due to optimization purposes in step 3, the Kappa values are converted to "1-Kappa" in the following sections.

Step 3: We estimate the trade-off point based on the three kinds of statistics within three dimensions in the 3D array. The desired generalization capacity should be achieving high performance of accuracy and diversity by maximizing two dimensions of zero-shot capabilities and robustness, while minimizing the dimension of model size as much as possible.

Searching the trade-off point over the 3D array (3*DA*) is described as,

3 $$\begin{aligned} \begin{aligned} \underset{(x,y,z) \in 3DA}{\min }\left( M_g(x, y, z)+SD_g(x, y, z)+{^{10}P_g(x, y, z)} +M_k(x, y, z)+SD_k(x, y, z)+{^{10}P_k(x, y, z)} \right) \\ \text{ subject } \text{ to } \left\{ \begin{array}{c} c_1: x \geqslant ZeroShot_{\min } \\ c_2: y \geqslant Robust _{\min } \\ c_3: z \leqslant WeightNum_{\max }\\ \end{array} \right. \end{aligned} \end{aligned}$$

where $(ZeroShot_{\min }, Robust_{\min }, WeightNum_{\max })$ are the given maximum(/minimum) bounds of three dimensions. Particularly, these bounds can be rewritten in an upper bound way. Equation (3) may be rewritten as follows,

4 $$\begin{aligned} \begin{aligned} \underset{(x,y,z,c_1,c_2,c_3)}{\min }\left( M_g(x, y, z)+SD_g(x, y, z)+{^{10}P_g(x, y, z)} +M_k(x, y, z)+SD_k(x, y, z)+{^{10}P_k(x, y, z)} \right) + \lambda \left\| C \right\| ^2 \\ \text{ subject } \text{ to: } \left\{ \begin{array}{c} c_1 \geqslant 1-x \\ c_2 \geqslant y \\ c_3 \geqslant z \\ \end{array} \right. \end{aligned} \end{aligned}$$

where $\lambda$ is a balancing coefficient and $C=(c_1,c_2,c_3)$ denotes the upper bounds. Ideally, the resulting (*x*, *y*, *z*) would be equal to the resulting $(c_1,c_2,c_3)$. We always select the resulting (*x*, *y*, *z*) as the trade-off point in practice.

To visualize it, we compute the marginal distributions with respect to three dimensions separately. The marginal distributions with respect to the dimension of *ZeroShot* is computed as,

5 $$\begin{aligned} \left\{ \begin{array}{c} M_g(x \sim 3 D A( ZeroShot ))= \sum _{(y, z) \sim 3 D A( Robust,WeightNum )} M_g(x, y, z) \\ SD_g(x \sim 3 D A( ZeroShot ))= \sum _{(y, z) \sim 3 D A( Robust,WeightNum )} SD_g(x, y, z) \\ {^{10}P_g(x \sim 3 D A( ZeroShot ))}= \sum _{(y, z) \sim 3 D A( Robust,WeightNum )} {^{10}P_g(x, y, z)} \\ M_k(x \sim 3 DA ( ZeroShot ))= \sum _{(y, z) \sim 3 D A( Robust,WeightNum )} M_k(x, y, z) \\ SD_k(x \sim 3 DA ( ZeroShot ))= \sum _{(y, z) \sim 3 D A( Robust,WeightNum )} SD_k(x, y, z) \\ {^{10}P_k(x \sim 3 DA ( ZeroShot ))}= \sum _{(y, z) \sim 3 D A( Robust,WeightNum )} {^{10}P_k(x, y, z)} \end{array}\right. \end{aligned}$$

There are a total of three sets of marginal distributions separately for three dimensions. Each set illustrates the generalization bounds (referred to as $M_g, SD_g, {^{10}P_g}$) and diversity (referred to as $M_k, SD_k, {^{10}P_k}$) concerning the scale at each dimension specified by the trade-off point, one after another. Theoretical equivalence is expected among these three sets of marginal probabilities at the trade-off point.

In fact, the trade-off point indicates the model’s tolerance on three dimensions at an expected marginal probability level. The area delimited by the trade-off point intuitively and quantitatively illustrates the generalization capacity of the test model. The trade-off point focuses on models with varying architectures rather than a single model.

## Results

We organise our experiments to illustrate how to determine the Trade-off points by the proposed practical generalisation metric, and then verify the existing complexity measures through the practical measurements based on our testbed. We hope to point out that the proposed benchmark testbed serves solely as an experimental platform to validate existing complexity measures.

### Data and test models

We use CIFAR-100 and ImageNet datasets^39^ for fine-tuning and tests. In our experiments, we pick up 50 classes for training and the rest 50 classes for the zero-shot scenario tests from CIFAR-100. We randomly select 100 object classes from ImageNet. Similarly, we divide it into two parts, i.e., 50 classes for training and the other 50 classes for tests. These two datasets are widely used in deep learning applications. The primary difference is the image size; ImageNet images are larger than those in CIFAR-100. Larger images in ImageNet provide more data, which generally leads to better learning outcomes. In contrast, the smaller images in CIFAR-100 often result in ambiguity, where additional context is necessary to accurately interpret the images. In addition, we apply augmentation approaches to these datasets to generate unseen data or classes in case that the pretrained models have seen data in their previous training.

We select the CLIP and EfficientNet models for benchmarking tests since they both share similar architecture. They have some connections as well as differences. We use 5 pre-trained CLIP models from Radford et al.^28^ and 8 EfficientNet models from Tan et al.^40^. Table 1 shows the pre-trained model sizes of CLIP and EfficientNet respectively. Although these pre-trained models have been optimised, they still need to be fine-tuned with the linear probe on the training data in advance. We only use the weight number of each model as the dimension of model size in the experiments, neglecting the other issues such as layers, depth, the change of structure, so that the pre-trained models line up in an “over-parameterization” way. We hope to have an insight to the generalisation capacity of these two kinds of pre-trained models, i.e. CLIP group and EfficientNet group. Moreover, the test data is added noises for robustness tests. To quantify noise levels, we employ the Autoencoder to the test data to generate noisy data and use the Structural SIMilarity (SSIM) Index metric to control noise levels. When SSIM is decreasing towards zero, the noise level is increasing. All the experiments work on a Workstation with Nvidia 12G RTX2080.

**Table 1:** Pretrained models’ parameters. The models include different variations of EfficientNet (B0-B7) and CLIP-based architectures, such as RN50, RN101, and vision transformer (ViT) models. These models are evaluated for their generalization performance in our benchmarking testbed.

EfficientNet	# Params	CLIP	# Params
efficientnet-b0	5.3M	RN50	38M
efficientnet-b1	7.8M	RN101	56M
efficientnet-b2	9.2M	RN50x4	87M
efficientnet-b3	12M	RN50x16	167M
efficientnet-b4	19M	RN50x64	420M
efficientnet-b5	30M	ViT-B/32	87M
efficientnet-b6	43M	ViT-B/16	86M
efficientnet-b7	66M	ViT-L/14	304M

### Trade-off points of CLIP and EfficientNet

The pre-trained CLIP models (i.e. RNxxx) and EfficientNet models are CNN-based (see Table 1). For comparison, the CLIP ViT-xxx models are not taken into account here.

Step 1: Collect ErrorRate and Kappa data of both kinds of test models

We test the pretrained models of CLIP and EfficientNet on test data across three dimensions (i.e., zero-shot%, weight number, SSIM) and store the error rates and Kappas for each class in each cell of a 3D array.

Step 2: Update 3D array

We compute three kinds of statistics related to the distributions of ErrorRate and Kappa across all classes, i.e., means, standard derivations, 10th percentiles, and update them cell-wise in the 3D array.

Step 3: Trade-off point

**Fig. 2:**
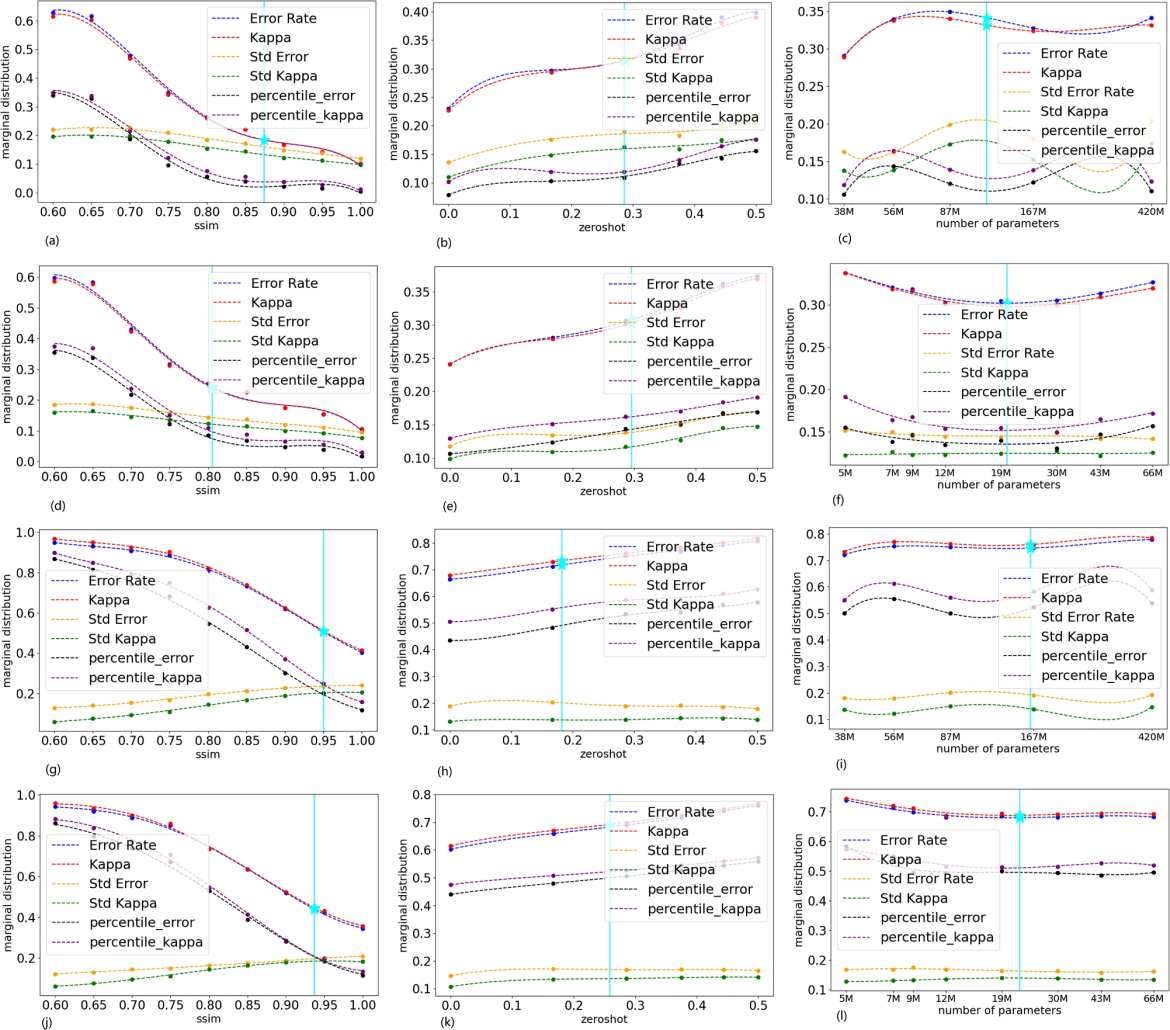
TradeOff points of two kinds models, CLIP and EfficientNet (denoted as “$\star$”). The solid vertical lines indicate the selection of trade-off points on each marginals. (**a**–**c**) CLIP on ImageNet, (**d**–**f**) EfficientNet on ImageNet, (**g**–**i**) CLIP on CIFAR-100, (**j**–**l**) EfficientNet on CIFAR-100. Neither CLIP nor EfficientNet consistently dominates across all conditions. While EfficientNet achieves better robustness and lower error rates on ImageNet, CLIP performs slightly better in zero-shot scenarios on CIFAR-100.

We compute the trade-off points by Eq. (4) and visualize the trade-off points by Eq. (5) based on three pairs of marginal distributions, as shown in Fig. 2. The trade-off points of CLIP and EfficientNet on CIFAR1-100 and ImageNet respectively are shown in Table 2.

**Table 2:** TradeOff points on ImageNet and CIFAR100. It can be noted that, the CLIP model does not outperform the EfficientNet model and the results on ImageNet are always better than on CIFAR-100.

Dataset	ImageNet		CIFAR-100	
MODEL TYPE	CLIP	EFFICIENT NET	CLIP	EFFICIENT NET
GENERALIZATION BOUND	0.279	0.284	0.657	0.600
DIVERSITY BOUND	0.276	0.280	0.668	0.608
SSIM (lower bound)	0.874	0.805	0.949	0.937
ZEROSHOT (upper bound)	0.285	0.295	0.182	0.258
MODEL SIZE (lower bound)	116M	20M	163M	22M

It can be noted that, (1) CLIP model does not outperform the EfficientNet model. Comparing the trade-off points in Table 2, CLIP’s generalization bound exceeds EfficientNet’s by up to 0.057 on CIFAR-100, and its diversity bound exceeds by up to 0.06. However, on ImageNet, CLIP’s generalization bound is lower by up to 0.005, and its diversity bound is lower by up to 0.004. We further compare three dimensions. The EfficientNet’s SSIM(lower bound), ZeroShot(upper bound) and Model size (lower bound) performance surpass that of CLIP. Particularly, EfficientNet’s model size is much smaller than CLIP’s.

**Fig. 3:**
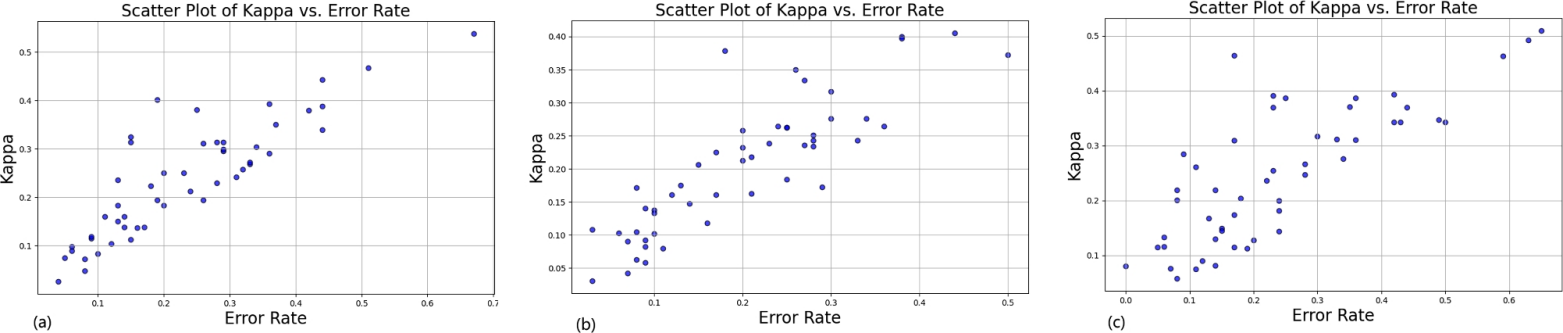
Scatter plot of Kappa vs. error rate under various settings (**a**) a CLIP model with SSIM = 1, ZEROSHOT = 20, Model Size = 38M, (**b**) a EfficientNet model with SSIM = 1, ZEROSHOT = 20, Model Size = 5.3M, (**c**) a CLIP model with SSIM = 0.8, ZEROSHOT = 30, Model Size = 167M.

Comparing the marginal distributions in Fig. 2, the trends of CLIP and EfficientNet (including ErrorRate and Kappa) across the SSIM, ZeroShot, and WeightNum dimensions are similar. We can further measure the diversity by ErrorRate and Kappa, that is, they can be visualized using the well-known “Kappa-Error diagrams” in a scatter plot, as shown in Fig. 3. In these diagrams, the distributions of error rates and Kappas across all classes form pairwise measures.

**Fig. 4:**
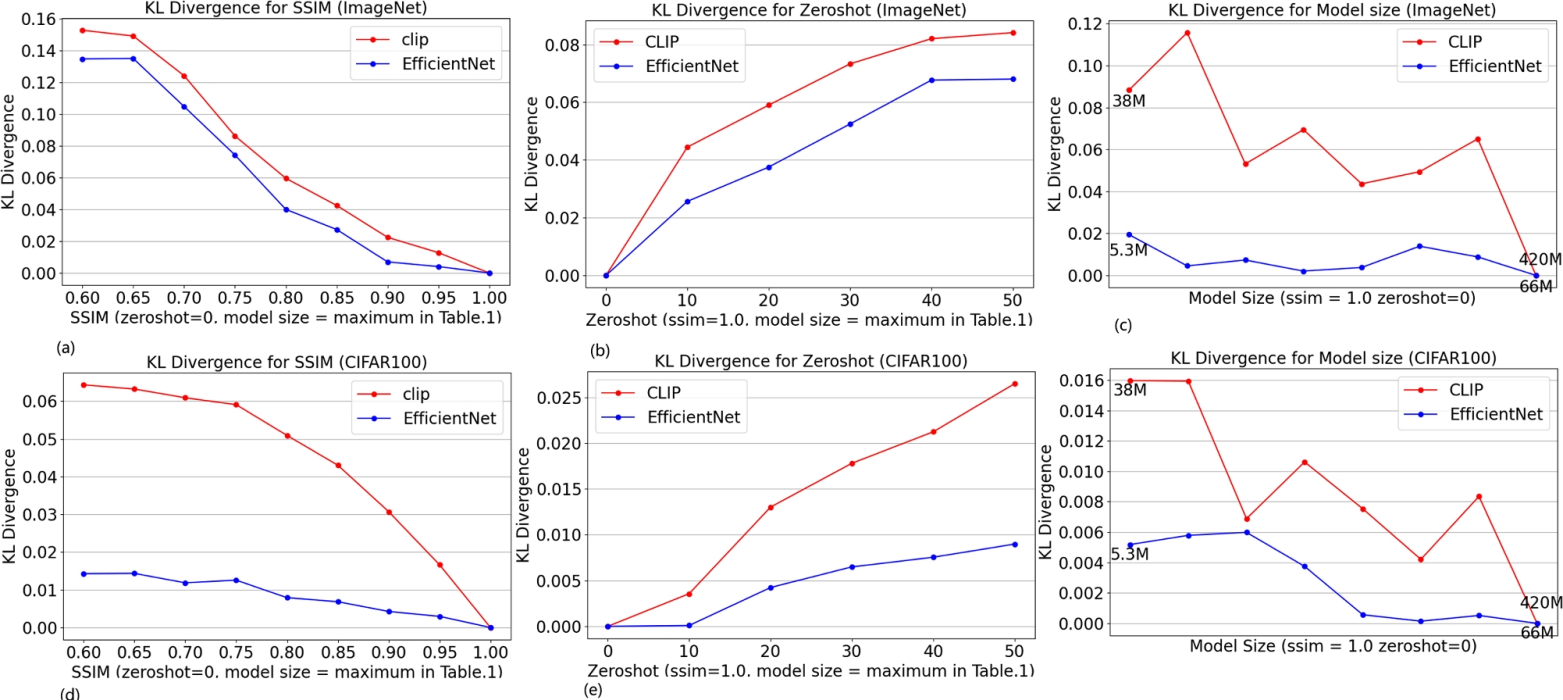
KL divergence measures the difference in performance distributions across different model dimensions (SSIM, ZeroShot, and Model Size). (**a**–**c**) KL divergence analysis for models on ImageNet. (**d**–**f**) KL divergence analysis for models on CIFAR-100. Lower divergence values indicate more stable generalization behavior across conditions.

For different dimensions (e.g., ZeroShot, Robust, WeightNum), numerous such pairwise measures exist. To further analyze these pairwise measures, we compute their KL divergence, as shown in Fig. 4. The KL divergences quantitatively capture the distance of model performance across different dimensions from the origin. As the distance increases, the performance of the model decreases. It can be noted that EfficientNet’s KL divergences are obviously less than CLIP’s.

Consequently, the CLIP model does not demonstrate a clear advantage against the EfficientNet model. A possible explanation is that the CLIP model is trained on a diverse set of (image, text) pairs, whereas our benchmarking does not include text as input. As a result, its performance is diminished.

(2) Difference between data sets. It can be noted that the generalisation and diversity bounds on ImageNet are much less than on CIFAR-100 in Table 2. Moreover, it can be noted that Error rates and Kappas on CIFAR-100 are obviously more than those on ImageNet in Figure 2. This indicates that the results on ImageNet are always better than on CIFAR-100 since big images can provide more data.

**Fig. 5:**
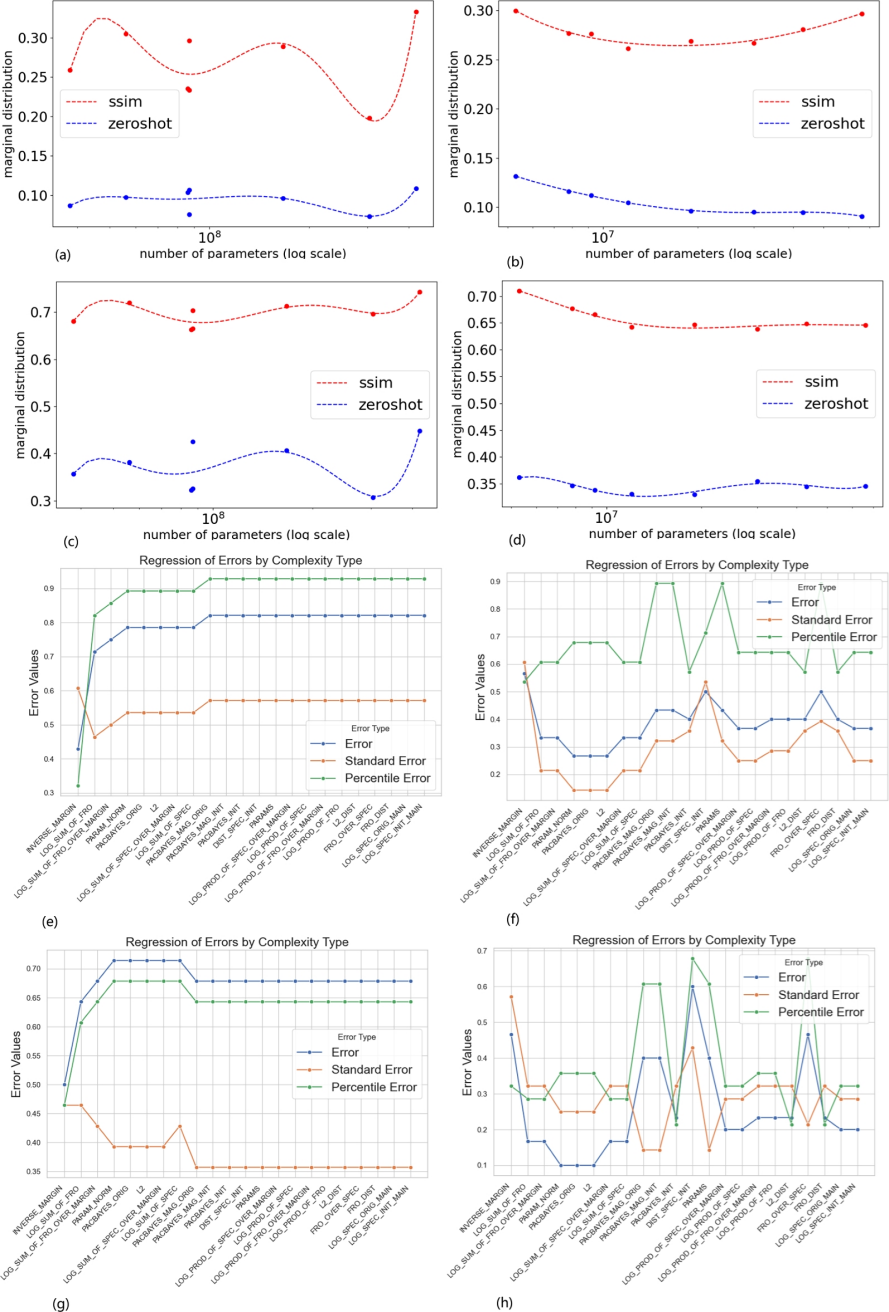
The upper four visualizes the marginal probability distributions of error rates in relation to model size (WeightNum) for two dataset slices: one with robustness and another without zero-shot capacity. (**a**) CLIP models on ImageNet. (**b**) EfficientNet models on ImageNet. (**c**) CLIP models on CIFAR-100. (**d**) EfficientNet models on CIFAR-100. The lower four presents scatter plots of sign-errors (SEg), which measure the inconsistency between theoretical complexity estimates and actual generalization behavior. (**e**) SEg for SSIM on ImageNet. (**f**) SEg for ZeroShot on ImageNet. (**g**) SEg for SSIM on CIFAR-100. (**h**) SEg for ZeroShot on CIFAR-100. Higher SEg values indicate larger discrepancies between theoretical generalization bounds and empirical measurements.

### Consistency check with existing generalisation estimations

Dziugaite et al.^2^ and recent work^4^ present 23 generalization measures, which we apply to all the pre-trained models listed in Table 1. Our goal is to assess the consistency between existing theoretical estimations and actual measures, and to evaluate agreement/disagreement rates among the available theoretical approaches. For comparison, we focus on two slices of the 3D array rather than the entire array: one for data without robustness and another for data without zero-shot capacity (see the pink and blue sections in Fig. 1b). This allows us to obtain two distributions of error rates-one for robustness and model size dimensions, and the other for zero-shot and model size dimensions. Note that Kappa is not considered here, as the available complexity estimations focus on generalization error rates. We conduct the consistency check between theoretical estimations and actual measures using these two distributions.

The dimensions of robustness and zero-shot capacity are regarded as two independent factors. We compute two marginal probabilities of these two slices with respect to the dimension of *WeightNum* (i.e., distributions with respect to *WeightNum*) as below,

6 $$\begin{aligned} \left\{ \begin{array}{c} dtr_g(z \sim 2DSLICE( WeightNum ))= \sum _{(y) \sim 2DSLICE( Robust )} dtr_g(y, z) \\ dtr_g(z \sim 2DSLICE( WeightNum ))= \sum _{(x) \sim 2DSLICE( ZeroShot )} dtr_g(x, z) \end{array}\right. \end{aligned}$$

Figure 5a–d shows these marginals based on ImageNet and CIFAR-100 respectively. Then, we compute the empirical sign-error of generalization in terms of the resulting marginal probabilities Eq. (6) as below,

7 $$\begin{aligned} \begin{array}{c} SE_{g} = \frac{1}{2}\mathbb {E}_{(w,w')\sim \left\{ WeightNum \right\} } \left[ 1-sgn(dtr_{g}(w) -dtr_{g}(w'))sgn(C(w)-C(w')) \right] \end{array} \end{aligned}$$

where *w* and $w'$ denote two different *WeightNum*s from the range of model size; *C*(.) denotes the complexity measures computed using Dziugaite et al.^2,4^. If the practical measures ($dtr_g$) and complexity measures (*C*) exhibit consistent changes, the sign-error ($SE_g$) approaches zero. Conversely, inconsistent changes lead to an $SE_g$ approaching one. Consequently, an $SE_g$ exceeding 0.5 indicates a potential mismatch between theoretical estimation and actual measures. Figure 5e–h visualizes the distributions of sign-errors through scatter plots.

**Table 3:** Top three complexity estimations. Among the 23 theoretical complexity measures tested, only a few exhibit moderate alignment with empirical generalization performance. This highlights a significant concern regarding the reliability of the theoretical estimation.

	Complexity measures	ImageNet		CIFAR-100	
		$SE_g$ of generalization bounds	$SE_g$ of 10th percentile	$SE_g$ of generalization bounds	$SE_g$ of 10th percentile
ZeroShot%	INVERSE_MARGIN	0.428571	0.321429	0.500000	0.464286
	LOG_SUM_OF_FRO	0.247638	0.163783	0.642857	0.607143
	PARAM_NORM	0.366667	0.392857	0.714286	0.678571
	p-value	$4.718e-10$		$8.786e-10$	
SSIM	INVERSE_MARGIN	0.285714	0.142857	0.466667	0.321429
	LOG_SUM_OF_FRO	0.714286	0.821429	0.166667	0.285714
	PARAM_NORM	0.785714	0.892857	0.10	0.357143
	p-value	$1.207e-10$		$1.504e-10$	

It can be noted that most of generalisation bound estimations are not consistent with actual measures. Regarding the robustness dimension (SSIM), although Fig. 5e shows that $30\%$ of $SE_g$ error rates exceed 0.5, Fig. 5g indicates that all $SE_g$ values are above 0.5. Additionally, in both Fig. 5e, g, the $SE_g$ values for the 10th percentile are greater than 0.5, implying that the top-performing $10\%$ of cases have an error rate exceeding $50\%$. This highlights a significant concern regarding the reliability of the estimation.

For the ZeroShot dimension, Fig. 5f shows that $43\%$ of $SE_g$ error rates exceed 0.5, whereas Fig. 5h shows that only $21\%$ exceed 0.5. This suggests that the estimation performs better in the ZeroShot dimension compared to robustness. However, in both Fig. 5f, h, most $SE_g$ values for the 10th percentile still exceed 0.3.

We ranked the complexity measures based on their performance in Fig. 5e–h and selected the top three: INVERSE_MARGIN, LOG_SUM_OF_FRO, and PARAM_NORM, as shown in Table 3. The smallest $SE_g$ values of the generalisation error bound and the 10th percentile are 0.25 and 0.14, respectively, indicating that the best-performing cases have error rates between $15\%$ and $25\%$.

In fact, the Sign-Error may be seen as a measure of bias across all complexity measures. We calculated p-values for the ZeroShot and SSIM dimensions based on all generalisation bounds in Fig. 5e-h, finding that all p-values are less than $10^{-10}$ (significantly below 0.05). This suggests a strong bias in the generalisation bounds, raising concerns about the reliability of these estimations.

## Conclusion

This paper introduces a practical generalization metric for benchmarking diverse deep networks and presents a novel testbed to validate theoretical estimations empirically. By identifying a quantifiable trade-off point, we establish a reliable indicator of deep network generalization capacity. Our results show a misalignment between existing generalization theories and our practical measurements.

### Limitations

This paper focuses on CLIP (CNN-based) and EfficientNet models, which limits the scope of the analysis. To enhance benchmarking, it is necessary to include a broader range of architectures. Additionally, our benchmarking considers three factors: model size, robustness, and zero-shot capabilities. However, these factors alone are insufficient to fully understand the architecture and behavior of the models.

### Future work

We plan to incorporate explainable AI tools, such as SHAP (SHapley Additive Explanations) and LIME (Local Interpretable Model-agnostic Explanations), into our benchmarking framework. Furthermore, we have initiated a public GitHub repository for deep network benchmarking. We encourage contributions to expand the dataset and promote further theoretical and practical research in the field. Furthermore, we will organise a comprehensive generalization benchmarking competition for deep networks. This future endeavor seeks to provide developers with a baseline platform to test new theories, thereby enhancing the understanding of why deep neural networks generalize. The benchmarking testbed will facilitate rigorous analyses, enabling developers to assess how well these theories align with the complexities observed in real-world models.

## Data Availability

All the data, models, and benchmarking results are available on GitHub (https://github.com/MENGQING912/A-practical-generalization-metric-for-deep-networks-benchmarking).
